# Risk Factors Associated with Unsuccessful Dofetilide Initiation Due to Excessive QT Interval Prolongation: A Retrospective Study

**DOI:** 10.19102/icrm.2025.16042

**Published:** 2025-04-15

**Authors:** Johnathon Rast, Grant Whitebloom, Omar M. Makram, Priyanshu Nain, Lakshya Seth, Nathaniel Wayne, Patrick Houlihan, Alexander Warner, Daniel Sohinki

**Affiliations:** 1 Department of Medicine, Medical College of Georgia at Augusta University, Augusta, GA, USA; 2 Division of Cardiology, Department of Medicine, Medical College of Georgia at Augusta University, Augusta, GA, USA

**Keywords:** Atrial fibrillation, dofetilide, QTc prolongation, torsades de pointes

## Abstract

Dofetilide is a class III anti-arrhythmic medication approved for patients with atrial fibrillation to maintain sinus rhythm. Excessive QTc interval prolongation, a potential side effect of dofetilide, increases the risk of torsades de pointes. This risk is mitigated by closely monitoring the QTc interval during an inpatient initiation protocol for the first five doses. Prior studies have demonstrated that dofetilide can be safely used in patients with heart failure after completing the initiation protocol. However, no studies have investigated risk factors associated with dofetilide-induced excessive QTc interval prolongation, resulting in discontinuation of the medicine. This single-center retrospective cohort study analyzed the association between dofetilide-associated excessive QTc prolongation during medication initiation and pertinent medical comorbidities as well as various echocardiographic values of interest. Risk factors found to be significantly associated with excessive QTc prolongation during dofetilide initiation included a clinical history of heart failure, reduced left ventricular ejection fraction, increased left ventricular end-diastolic diameter, increased left atrial diameter, and reduced right ventricular systolic function. Although some studies have demonstrated the safety of dofetilide use in patients with heart failure, our findings suggest that these patients are less likely to tolerate initiation of the medication due to excessive QTc prolongation.

## Introduction

Dofetilide, a class III anti-arrhythmic drug, is approved for rhythm control in patients with atrial fibrillation (AF) who remain symptomatic with a rate-control strategy.^1–5^ Several randomized studies have demonstrated dofetilide to be a safe and effective option for patients with symptomatic AF or atrial flutter.^2–4^ However, this medication is associated with an increased incidence of torsade de pointes (TDP) because of its potential to prolong the QT interval.^2–4,6^ The frequency of dofetilide-induced TDP has been demonstrated to be highest within the first 3 days of dofetilide use.^3,5^ Thus, patients initiating dofetilide must be admitted to the hospital for frequent QT interval monitoring to safely load the first five doses.^2–4^ If the QT interval corrected for heart rate (QTc) is prolonged to >500 ms after the second dose of dofetilide during initiation, the medication must be discontinued due to the elevated risk of developing the life-threatening arrhythmia TDP. The Danish Investigations of Arrhythmia and Mortality on Dofetilide in Congestive Heart Failure (DIAMOND-CHF) study demonstrated that patients with heart failure were not at excessively high risk of developing TDP when the medication was appropriately initiated and renally dosed.^3^ However, they also found that women and patients classified as having New York Heart Association (NYHA) class III or IV symptomatic heart failure were significantly more likely to develop dofetilide-associated TDP.^3,6,7^

To the best of our knowledge, no studies have investigated factors that may increase the risk of excessive QT prolongation during initiation, requiring discontinuation of dofetilide. Our goal was to investigate the association between dofetilide-induced excessive QT prolongation during dofetilide initiation and objective echocardiographic measurements as well as medical comorbidities. Initiating dofetilide carries upfront costs and risks. Patients who must discontinue dofetilide due to a significantly prolonged QTc interval during initiation are briefly exposed to the risk of TDP. In fact, a significant portion of TDP episodes occur during the first few doses.^3,6^ Patients who must discontinue dofetilide are also burdened with the cost and time associated with a multiday hospital stay for an ultimately unsuccessful treatment strategy. These costs and risks warrant investigation into risk factors associated with unsuccessful dofetilide initiation to optimize patient selection for dofetilide therapy.

## Methods

### Study setting and data source

This retrospective cohort study was conducted at a single-center tertiary care hospital. The electronic medical records of all patients at the study medical center who underwent dofetilide initiation protocol between January 2004 and May 2022 were screened for candidacy.

This study was approved by the institutional review board of the involved medical center. The need for patient consent was waived due to the retrospective nature of the study. All involved researchers followed appropriate protocols for secure data handling of patient medical information.

### Study cohort and conduction

Patients were included if they underwent standard dofetilide initiation protocol and had a transthoracic echocardiogram (TTE) within 2 years prior to dofetilide initiation. The dofetilide initiation protocol considered standard in this study was the Symptomatic Atrial Fibrillation Investigative Research on Dofetilide (SAFIRE-D) study protocol,^2^ which is as follows: patients with a baseline QTc of <440 ms and creatinine clearance (CrCl) of >20 mL/min were considered for dofetilide therapy. Initial dosing increments included 500 μg every 12 h for CrCl > 60 mL/min, 250 μg every 12 h for CrCl 40–60 mL/min, and 125 μg every 12 h for CrCl 20–39 mL/min. If the QTc is increased by >15% on an electrocardiogram (ECG) performed 2–3 h after the first dose, the subsequent dose is decreased to the next lowest available dose. If the starting dose was 125 μg every 12 h, the subsequent doses would be given every 24 h. If the QTc prolonged to >500 ms after any subsequent dose, dofetilide was discontinued. The QTc was monitored on continuous telemetry and with 12-lead ECGs recorded 2–3 h after each of the first five doses. If the QTc remained <500 ms after five doses and after following the above dosing adjustments, patients were successfully initiated on dofetilide and considered safe to continue the medication.^2^ Prior to starting the initiation protocol, all contraindicated medications were discontinued, and other QT-prolonging medications considered non-essential were similarly discontinued. Daily laboratory samples were collected to measure serum potassium and magnesium levels, which were repleted to goal levels of 4 mEq/L and 2 mg/dL, respectively. An exception was made to this protocol for patients with ventricular paced rhythms—a baseline QTc of <440 ms was not considered necessary for them to start dofetilide. In these patients, the cardiac electrophysiology providers monitored the JTc interval during dofetilide initiation instead of the QTc interval. If the JTc interval ever prolonged >15% above the baseline JTc, the dofetilide dose or frequency was reduced. If any repeat ECGs showed persistence or recurrence of JTc prolongation by >15% above the baseline JTc, dofetilide was stopped and initiation was considered unsuccessful. This strategy was used because a prolonged QRS complex in ventricular paced rhythms causes baseline QTc prolongation, which interferes with the reliability of using traditional QTc value cutoffs of normal conduction for determining increased TDP risk.^8^ Additionally, tracking JTc interval changes during anti-arrhythmic drug initiation appears to be a superior method for detecting unsafe repolarization changes compared to tracking QTc interval changes in patients receiving ventricular pacing.^8^ Patients were excluded if dofetilide initiation was stopped for any reason other than excessive QTc or JTc prolongation, such as unsuccessful chemical cardioversion with dofetilide, medication affordability concerns, non-cardiac intolerable side effects, or significant deviation from the standard dofetilide initiation protocol.

### Risk factors

Variables collected included patient demographics (age, sex), pertinent medical comorbidities (congestive heart failure with NYHA classification, coronary artery disease, diabetes mellitus, hypertension, chronic kidney disease, chronic obstructive pulmonary disease, obstructive sleep apnea), certain TTE variables of interest (left ventricular ejection fraction [LVEF], left atrial [LA] diameter, left ventricular end-diastolic diameter, right atrial diameter, right ventricular [RV] systolic function, RV size, aortic stenosis severity, aortic regurgitation severity, mitral regurgitation severity, tricuspid regurgitation severity, pulmonic regurgitation severity), and all ECG studies during dofetilide initiation. The ECGs analyzed included a baseline ECG recorded shortly before dofetilide initiation as well as an ECG recorded approximately 2–3 h after each of the first five dofetilide doses. Data collected from each ECG included the heart rate, underlying rhythm, QRS duration, QT interval, and QTc interval. In patients with ventricular pacing, JT and JTc intervals were also collected. The QTc intervals were manually measured and corrected for the heart rate with Fridericia’s formula. Fridericia’s formula was selected due to its superior QTc measuring precision at higher and lower heart rates,^9^ thus improving data precision when analyzing ECGs with significant tachycardia or bradycardia.

### Statistical analysis

Patient characteristics were presented using absolute numbers and percentages for categorical variables and using median and interquartile range (IQR) values for continuous variables after confirming the non-normality of the data visually and using the Shapiro–Wilk test. For categorical variables, the chi-squared test was conducted to compare the categorical risk factors between the individuals who passed and those who failed the initiation protocol. For non-normally distrusted continuous variables, the Mann–Whitney *U* test was conducted, and *P* values were estimated. A comprehensive risk factor analysis with a multivariable logistic regression model was conducted to quantify the odds of dofetilide initiation failure for each potential risk factor. In addition to an unadjusted statistical model, a set of pre-specified variables was used to create the multivariable model 1, which was adjusted for biologic sex and age. Statistical analysis was performed using STATA/IC 16.1 (StataCorp, College Station, TX, USA). Results were considered statistically significant if the calculated *P* value was <.05.

## Results

A total of 223 patients were included in the study, of whom 166 (74.4%) were successfully initiated on dofetilide without excessive QTc prolongation (>500 ms) **(Table 1)**. The primary endpoint, QTc prolongation to >500 ms any time between dofetilide doses 2 and 5, occurred in 57 (25.6%) patients. Patient demographics, medical comorbidities, and the occurrence of the primary endpoint are illustrated in **Table 1**. Female sex as well as NYHA III and IV symptomatic heart failure are known risk factors for dofetilide-associated TDP,^3^ but they were not significantly associated with unsuccessful dofetilide initiation in this study (women: odds ratio [OR], 1.80; 95% confidence interval [CI], 0.98—3.31; *P* = .059) (NYHA III and IV: OR, 1.28; 95% CI, 0.47–3.48; *P* = .63) **(Table 2)**.

**Table 1:** Demographics, Past Medical History, and Transthoracic Echocardiography Parameters of the Included Individuals

Characteristics		Total	Successful	Unsuccessful	*P* Value*
Sample size, n (%)		221	164 (74.2%)	57 (25.8%)	
Baseline QTc, median (IQR)		412.0 (389.0–437.5)	406.0 (386.0–430.0)	422.0 (402.0–450.0)	.006**
Maximum QTc, median (IQR)		463.0 (440.0–496.0)	452.0 (434.0–474.0)	519.5 (505.0–542.0)	<.001**
Demographic factors					
Age (years), median (range)		67.0 (60.0–74.0)	67.0 (60.0–74.0)	66.0 (60.0–73.0)	.94
Sex, n (%)	Male	117 (52.9%)	93 (56.7%)	24 (42.1%)	.057
	Female	104 (47.1%)	71 (43.3%)	33 (57.9%)	
Existing cardiovascular risk factors and diseases					
CAD, n (%)		86 (38.9%)	62 (37.8%)	24 (42.1%)	.57
HF, n (%)		86 (38.9%)	47 (28.7%)	39 (68.4%)	<.001**
Diabetes, n (%)		55 (24.9%)	36 (22.0%)	19 (33.3%)	.087
Hypertension, n (%)		181 (81.9%)	133 (81.1%)	48 (84.2%)	.60
CKD, n (%)		45 (20.4%)	38 (23.2%)	7 (12.3%)	.079
COPD, n (%)		18 (8.1%)	12 (7.3%)	6 (10.5%)	.45
OSA, n (%)		33 (14.9%)	23 (14.0%)	10 (17.5%)	.52
NYHA class, n (%)	I–II	66 (76.7%)	37 (78.7%)	29 (74.4%)	.63
	III	20 (23.3%)	10 (21.3%)	10 (25.6%)	
Echo parameters					
LA diameter, n (%)	≤4 cm	88 (40.0%)	73 (44.8%)	15 (26.3%)	.014**
	>4 cm	132 (60.0%)	90 (55.2%)	42 (73.7%)	
LA diameter, median (IQR)		4.3 (3.8–4.9)	4.2 (3.7–4.8)	4.6 (4.0–5.1)	.011**
LVEF	<20%	13 (5.9%)	8 (4.9%)	5 (8.8%)	.005**
	20%–30%	16 (7.2%)	11 (6.7%)	5 (8.8%)	
	30%–40%	24 (10.9%)	15 (9.1%)	9 (15.8%)	
	40%–50%	24 (10.9%)	12 (7.3%)	12 (21.1%)	
	>50%	144 (65.2%)	118 (72.0%)	26 (45.6%)	
LVIDD, median (IQR)		4.6 (4.0–5.2)	4.6 (4.0–5.2)	4.8 (4.2–5.5)	.075
RA dilation, n (%)		73 (38.6%)	50 (35.5%)	23 (47.9%)	.13
RV reduced systolic function, n (%)		46 (25.1%)	29 (21.5%)	17 (35.4%)	.056
RV enlarged size, n (%)		77 (41.0%)	55 (39.3%)	22 (45.8%)	.43
Mitral regurgitation, n (%)	None/trace	64 (33.0%)	50 (35.0%)	14 (27.5%)	.050
	Mild	100 (51.5%)	76 (53.1%)	24 (47.1%)	
	Moderate	26 (13.4%)	16 (11.2%)	10 (19.6%)	
	Severe	4 (2.1%)	1 (0.7%)	3 (5.9%)	
Aortic regurgitation, n (%)	None/trace	148 (76.3%)	111 (77.6%)	37 (72.5%)	.67
	Mild	41 (21.1%)	29 (20.3%)	12 (23.5%)	
	Moderate	5 (2.6%)	3 (2.1%)	2 (3.9%)	
Aortic stenosis, n (%)	None/trace	178 (92.2%)	132 (93.0%)	46 (90.2%)	.23
	Mild	8 (4.1%)	7 (4.9%)	1 (2.0%)	
	Moderate	5 (2.6%)	2 (1.4%)	3 (5.9%)	
	Severe	2 (1.0%)	1 (0.7%)	1 (2.0%)	
Pulmonic regurgitation, n (%)	None/trace	147 (76.6%)	113 (80.1%)	34 (66.7%)	.15
	Mild	42 (21.9%)	26 (18.4%)	16 (31.4%)	
	Moderate	3 (1.6%)	2 (1.4%)	1 (2.0%)	
Tricuspid regurgitation, n (%)	None/trace	65 (33.5%)	50 (35.0%)	15 (29.4%)	.30
	Mild	102 (52.6%)	76 (53.1%)	26 (51.0%)	
	Moderate	22 (11.3%)	15 (10.5%)	7 (13.7%)	
	Severe	5 (2.6%)	2 (1.4%)	3 (5.9%)	

*Abbreviations:* CAD, coronary artery disease; CKD, chronic kidney disease; COPD, chronic obstructive pulmonary disease; HF, heart failure; LA, left atrium; LVEF, left ventricle ejection fraction; LV IDD, left ventricle internal diameter in diastole; OSA, obstructive sleep apnea; RA, right atrium; RV, right ventricle. *P value representing chi-squared test for categorical variables and the Mann–Whitney *U* test for continuous variables. **Statistically significant findings.

**Table 2:** Logistic and Linear Regression for the Relationship Between the Various Risk Factors and Unsuccessful Dofetilide Initiation

Risk Factors		Univariable Model	Model 1 (Each Variable Adjusted for Age and Sex)
		OR (95% CI; *P* Value)	aOR (95% CI; *P* Value)
**Logistic regression**			
Age		1.01 (0.98–1.04; *P* = .545)	1.005 (0.98–1.03; *P* = .712)
Female sex		1.80 (0.98–3.31; *P* = .059)	1.78 (0.96–3.28; *P* = .067)
Baseline QTc	For every 40-ms increase	1.01 (1.002–1.019; *P* = .016)*	1.01 (1.001–1.018; *P* = .024)*
	For every 10-mm increase	1.11 (1.02–1.21; *P* = .016)*	1.10 (1.01–1.20; *P* = .024)*
Maximum QTc		1.05 (1.04–1.07; *P* < .001)*	
CAD		1.19 (0.65–2.21; *P* = .566)	1.19 (0.63–2.23; *P* = .592)
HF		5.39 (2.81–10.36; *P* < .001)*	6.07 (3.08–11.98; *P* < .001)*
Diabetes		1.78 (0.92–3.45; *P* = .089)	1.94 (0.98–3.84; *P* = .058)
Hypertension		1.24 (0.55–2.80; *P* = .599)	1.25 (0.55–2.86; *P* = .591)
CKD		0.46 (0.19–1.11; *P* = .084)	0.47 (0.19–1.14; *P* = .094)
COPD		1.49 (0.53–4.17; *P* = .448)	1.47 (0.52–4.14; *P* = .471)
OSA		1.30 (0.58–2.94; *P* = .522)	1.55 (0.67–3.58; *P* = .307)
NYHA class	I–II	Reference	Reference
	III	1.28 (0.47–3.48; *P* = .634)	1.14 (0.40–3.20; *P* = .809)
LA diameter	≤4 cm	Reference	Reference
	>4 cm	2.27 (1.17–4.42; *P* = .016)*	2.91 (1.44–5.89; *P* = .003)*
LVEF	<40%	2.54 (1.25–5.13; *P* = .010)*	3.29 (1.55–7.02; *P* = .002)*
	40%–50%	4.54 (1.83–11.23; *P* = .001)*	5.07 (1.98–12.95; *P* = .001)*
	>50%	Reference	Reference
RA dilation		1.67 (0.86–3.25; *P* = .128)	1.84 (0.93–3.65; *P* = .081)
RV reduced systolic function		2.01 (0.98–4.12; *P* = .058)	2.26 (1.07–4.74; *P* = .032)*
RV enlarged size		1.31 (0.67–2.53; *P* = .427)	1.54 (0.76–3.09; *P* = .229)
Mitral regurgitation	None/trace	Reference	Reference
	Mild	1.13 (0.53–2.39; *P* = .753)	1.04 (0.48–2.25; *P* = .919)
	Moderate	2.23 (0.83–5.99; *P* = .111)	2.10 (0.77–5.70; *P* = .145)
	Severe	10.71 (1.03–111.17; *P* = .047)*	9.33 (0.88–98.46; *P* = .063)
Aortic regurgitation	None/trace	Reference	Reference
	Mild	1.24 (0.58–2.68; *P* = .581)	1.35 (0.61–2.98; *P* = .463)
	Moderate	2.00 (0.32–12.44; *P* = .457)	1.86 (0.29–11.78; *P* = .511)
Aortic stenosis	None/trace	Reference	Reference
	Mild	0.41 (0.05–3.42; *P* = .410)	0.36 (0.4–3.07; *P* = .351)
	Moderate	4.30 (0.69–26.58; *P* = .116)	4.32 (0.68–27.55; *P* = .121)
	Severe	2.87 (0.18–46.82; *P* = .459)	3.14 (0.19–53.04; *P* = .428)
Pulmonic regurgitation	None/trace	Reference	Reference
	Mild	2.05 (0.98–4.25; *P* = .055)	1.93 (0.92–4.06; *P* = .083)
	Moderate	1.66 (0.15–18.89; *P* = .682)	1.22 (0.10–14.29; *P* = .873)
Tricuspid regurgitation	None/trace	Reference	Reference
	Mild	1.14 (0.55–2.36; *P* = .724)	0.99 (0.47–2.14; *P* = .999)
	Moderate	1.56 (0.54–4.52; *P* = .417)	1.29 (0.43–3.87; *P* = .652)
	Severe	5.00 (0.76–32.77; *P* = .093)	4.19 (0.62–28.57; *P* = .142)
**Linear regression**			
LA diameter, mm		1.10 (1.02–1.18; *P* = .015)*	1.13 (1.05–1.22; *P* = .002)*
LVIDD, mm		1.06 (0.99–1.14; *P* = .071)	1.11 (1.03–1.19; *P* = .008)*

*Abbreviations:* aOR, adjusted odds ratio; CAD, coronary artery disease; CKD, chronic kidney disease; COPD, chronic obstructive pulmonary disease; HF, heart failure; LA, left atrium; LVEF, left ventricle ejection fraction; LVIDD, left ventricle internal end-diastolic diameter; OR, odds ratio; OSA, obstructive sleep apnea; RA, right atrium; RV, right ventricle. *Statistically significant findings.

The only medical comorbidity found to be significantly associated with an increased risk of unsuccessful dofetilide initiation was congestive heart failure, regardless of LVEF (OR, 5.39; 95% CI, 2.81–10.36; *P* < .001) (adjusted OR [aOR], 6.07; 95% CI, 3.08–11.98; *P* < .001). Several echocardiographic parameters were significantly associated with unsuccessful dofetilide initiation in the univariable model and the model adjusted for age and biologic sex, including LA diameter > 4 cm (OR, 2.27; 95% CI, 1.17–4.42, *P* = .016) (aOR, 2.91; 95% CI, 1.44–5.89; *P* = .003), LVEF < 40% (OR, 2.54; 95% CI, 1.25–5.13; *P* = .010) (aOR, 3.29; 95% CI, 1.55–7.02; *P* = .002), and LVEF 40%–49% (OR, 4.54; 95% CI, 1.83–11.23; *P* = .001) (aOR, 5.07; 95% CI, 1.98–12.95; *P* = .001). Reduced RV systolic function was found to be significantly associated with unsuccessful dofetilide initiation but only after adjustment for age and biologic sex (aOR, 2.26; 95% CI, 1.07–4.74; *P* = .032) **(Table 2)**.

Logistic regression revealed multiple significant findings. For every 1-mm increase in LA diameter, the unadjusted risk of unsuccessful dofetilide initiation increased by 10% (OR, 1.10; 95% CI, 1.02–1.18; *P* = .015) and the adjusted risk increased by 13% (aOR, 1.13; 95% CI, 1.05–1.22; *P* = .002). Every 1-mm increase in LV end-diastolic diameter increased the risk of unsuccessful dofetilide initiation by 11% when adjusted for age and biologic sex (aOR, 1.11; 95% CI, 1.03–1.19; *P* = .008). Additionally, every 40-ms increase in the baseline QTc interval increased the risk of unsuccessful dofetilide initiation by 1% (OR, 1.01; 95% CI, 1.002–1.019; *P* = .016) (aOR, 1.01; 95% CI, 1.001–1.018; *P* = .024) **(Table 2)**.

## Discussion

Dofetilide and amiodarone are the only AF rhythm-control agents thought to be safe for patients diagnosed with heart failure with reduced ejection fraction (HFrEF).^5^ Specifically, the Danish Investigations of Arrhythmia and Mortality on Dofetilide in Congestive Heart Failure (DIAMOND-CHF) study demonstrated that patients with heart failure on dofetilide who safely tolerated appropriate dofetilide initiation are not at significantly increased risk of arrhythmic deaths relative to the study placebo control group.^3^ This may be in part due to early detection of dofetilide-induced QTc prolongation and subsequent discontinuation of the medication. Research has demonstrated that a vast majority of patients who develop dofetilide-induced excessive QTc prolongation, and thus an increased risk of TDP, will do so within the first five doses.^3,5^ This early onset of QTc prolongation justifies the time and financial cost of admitting patients in the hospital for dofetilide initiation. To optimize patient selection for inpatient dofetilide initiation, it would be valuable to identify factors that may reduce the likelihood of successful dofetilide initiation. Until now, research into factors associated with dofetilide-induced QTc prolongation during dofetilide initiation has not been investigated directly. Our data suggest that a clinical history of heart failure, reduced LVEF < 50%, increased LA diameter, increased left ventricular end-diastolic diameter, reduced RV systolic function, and baseline QTc prolongation are all associated with higher rates of dofetilide-induced excessive QTc prolongation during dofetilide initiation.

The linear relationship between the severity of LA enlargement and likelihood of excessive QTc prolongation during dofetilide initiation is particularly interesting. Atrial structure alone has no known direct impact on the QT interval; however, LA enlargement is often a consequential effect of prolonged ventricular dysfunction.^10^ Thus, perhaps this relationship exemplifies an association between the duration and degree of ventricular dysfunction and the likelihood of excessive QTc prolongation during dofetilide initiation. This hypothesis is further supported by the direct association between increased left ventricular end-diastolic diameter and a greater likelihood of excessive QTc prolongation during dofetilide initiation. Our data suggest that, the larger the left ventricle becomes, the greater the likelihood of dofetilide-induced excessive QTc prolongation.

The DIAMOND-CHF study revealed that female sex was a significant risk factor for TDP in their studied population of patients, which only included patients with heart failure.^3^ Our retrospective study, which included patients with and without heart failure, found the influence of biologic sex to approach statistical significance (*P* = .059). Additionally, female sex is a known risk factor for medication-induced QTc prolongation and for TDP.^7,11–13^ Given these factors, we decided to adjust our statistical analysis for biologic sex. Although NYHA III–IV heart failure is a known risk factor for TDP for patients taking dofetilide,^3,6,7^ our study did not identify a significantly higher risk of dofetilide-associated QTc prolongation in these patients. This could be due to the small sample size of NYHA III (n = 6) and NYHA IV (n = 0) patients. Considering these factors and that many of our patients do not have heart failure, NYHA III–IV heart failure was not a component used in our adjusted statistical models. Older age is also a known risk factor for drug-induced QTc prolongation.^11,13–15^ Although age did not significantly differ between the successful and unsuccessful dofetilide-initiation groups in our data (see **Table 1**), we chose to adjust for age in our adjusted statistical model as it is a known risk factor.

Drug-induced QTc prolongation remains a partially unpredictable occurrence, but several factors have been identified to increase the risk of drug-induced QTc prolongation. Female sex is an established risk factor for drug-induced QTc prolongation, and overall TDP frequency is higher in women than in men.^7,11–13^ Structural heart disease, including HFrEF, is also a known risk factor for drug-induced QTc prolongation and TDP,^11,13–15^ which supports some of our study’s findings. Other risk factors for drug-induced QTc prolongation include advanced age, sepsis, the use of multiple QT-prolonging medications, active diuresis, bradycardia, and electrolyte abnormalities such as hypokalemia and hypomagnesemia.^11,13–15^ Our patient cohort had all unnecessary QT-prolonging medications discontinued and daily serum labs completed to guide the repletion of serum magnesium and potassium levels as needed.

Recently, one retrospective study found an association between dofetilide and sotalol-induced QTc prolongation in patients with HFrEF and heart failure with preserved ejection fraction compared to patients without heart failure.^16^ However, this was not studied exclusively in the setting of dofetilide initiation. Additionally, specific echocardiographic parameters associated with QTc prolongation during dofetilide initiation were not previously studied. Our data show that patients with HFrEF, or any clinical heart failure, may be at elevated risk for not safely tolerating dofetilide initiation when compared to the total AF population. Our study will hopefully provide further nuance into the risk–reward decision of admitting a patient in the hospital for dofetilide initiation. Patients with multiple risk factors for unsuccessful dofetilide initiation may be led down an alternative management option, optimizing efficiency for the health care system and providing financial and safety benefits for the patient.

### Limitations

Our study contains several limitations. A larger sample size may identify a greater number of risk factors for dofetilide-associated QT prolongation. Additionally, our single-center study weakens the generalizability of our findings. Our study also did not exclude or adjust for patients undergoing active diuresis, patients on other necessary QT-prolonging medications, patients with sinus bradycardia, and patients with other risk factors for excessive QTc prolongation. Our study allowed for the inclusion of TTE studies within a 2-year range prior to dofetilide initiation, but several echocardiographic values may change drastically during such a large timeline. Of 223 patients, 163 (73%) had a TTE within 6 months prior to dofetilide initiation. The longer 2-year timeline was chosen to endure adequate study power, but TTE studies performed at longer times prior to dofetilide initiation may offer less certainty regarding the precision of their recorded echocardiographic values. Our study contained a very small number of patients with NHYA class III (n = 6) heart failure and no patients with NYHA class IV heart failure. This weakens the generalizability of our findings to patients with these advanced symptomatic classes of heart failure.

## Conclusion

Our study suggests a direct association between the likelihood of dofetilide-induced excessive QTc prolongation and a clinical history of congestive heart failure, a reduced LVEF, increased LA diameter, and increased left ventricular end-diastolic diameter. Larger, multicenter studies are warranted to confirm these findings, which challenge the currently accepted notion that dofetilide safety is similar for patients with and without cardiomyopathy. Future studies may add further predictive value regarding risk factors for dofetilide-induced excessive QTc prolongation, aiding in optimal patient selection for dofetilide therapy.
